# Effective Low-Cost Ophthalmological Screening With a Novel iPhone Fundus Camera at Community Centers

**DOI:** 10.7759/cureus.28121

**Published:** 2022-08-17

**Authors:** Du Cheng, Rachel Babij, Daniel Cabrera, Melissa Yuan, Alexander Port, Anna Sophia Mckenney, Jeff Zhu, Sarah Van Tassel, Julianne Imperato-McGinley, Grace Sun

**Affiliations:** 1 Ophthalmology, NewYork-Presbyterian/Weill Cornell Medical Center, New York, USA; 2 Opthalmology, NewYork-Presbyterian/Weill Cornell Medical Center, New York, USA; 3 Psychiatry, NewYork-Presbyterian/Weill Cornell Medical Center, New York, USA; 4 Neurological Surgery, NewYork-Presbyterian/Weill Cornell Medical Center, New York, USA; 5 Ophthalmology, NJRetina, New Brunswick, USA; 6 Radiology, NewYork-Presbyterian/Weill Cornell Medical Center, New York, USA; 7 Clinical and Translational Science Center, NewYork-Presbyterian/Weill Cornell Medical Center, New York, USA

**Keywords:** training & education, telemedicine (tm), community-based, vision screening, glaucoma suspect

## Abstract

Ophthalmologic care is inaccessible to many people due to a variety of factors, including the availability of providers, cost of equipment for ophthalmologic care, and transportation to clinics and appointments. Because many causes of blindness are both highly prevalent and preventable once identified, it is essential to address gaps in care for underserved populations. We developed a novel 3D-printed mobile retinal camera. In this study, we organized recurring student-run screening events around New York City that took place in community centers and churches, at which we utilized our device to take retinal images. Our screening events reached a diverse population of New Yorkers, disproportionately those with lower household income, many of whom had not had recent eye exams. To validate the device for use in telehealth ophthalmologic visits, we transmitted the images to a remote ophthalmologist for evaluation and compared the result with an on-site attending physician’s dilated eye exam. The subjective assessment indicated that 97% of images captured with the mobile retinal camera were acceptable for telehealth analysis. Remote image assessment by achieved 92% sensitivity and 83% specificity in detecting optic disc cupping, compared to the gold-standard on-site dilated eye exam. In addition, the device was portable, affordable, and able to be used by those with relatively little ophthalmologic training. We have demonstrated the utility of this affordable mobile retinal camera for telehealth ophthalmologic evaluation during community screening events that reached an underserved population to detect disease and connect with long-term care.

## Introduction

A significant amount of blindness within the US and worldwide is preventable, though many populations lack access to ophthalmologic care due to a lack of health insurance and low health literacy [1,2]. Furthermore, early in the disease course, patients with ocular diseases frequently have minimal visual symptoms and will thus fail to present for evaluation. Screening and follow-up are often lacking in medically underserved areas [3,4], and patients in remote areas are often more likely to have inadequate awareness of ophthalmologic issues, such as glaucoma [5]. Community populations would therefore benefit from ophthalmological screening, including retinal examination by fundus imaging, to prevent irreversible loss of vision.

Barriers to proper ophthalmologic screening in underserved populations arise from various factors, including lack of access to personnel with appropriate expertise, transportation difficulties and inconvenience, cost of care/lack of insurance, and cost of equipment needed for ophthalmologic care [6,7]. A proper retinal fundoscopic examination typically requires significant expertise or retinal imaging with expensive equipment. An easy-to-use, affordable fundoscopic camera would allow for an expansion in ophthalmologic telehealthcare. In recent years, technological advances have created new opportunities to deliver much-needed ophthalmologic care to patients in remote areas [3,8-10], though the cost and availability of trained ophthalmologists remain significant issues. The primary aim of this study was to test the validity of an affordable, portable mobile retinal camera by comparing it with the conventional dilated fundus exam in a community setting. Focusing on remote fundus image assessment, we ask whether conducting ophthalmology screening in the community can identify participants with an increased cup to disk ratio that suggests undiagnosed glaucoma.

## Materials and methods

Screening events

**Figure 1:**
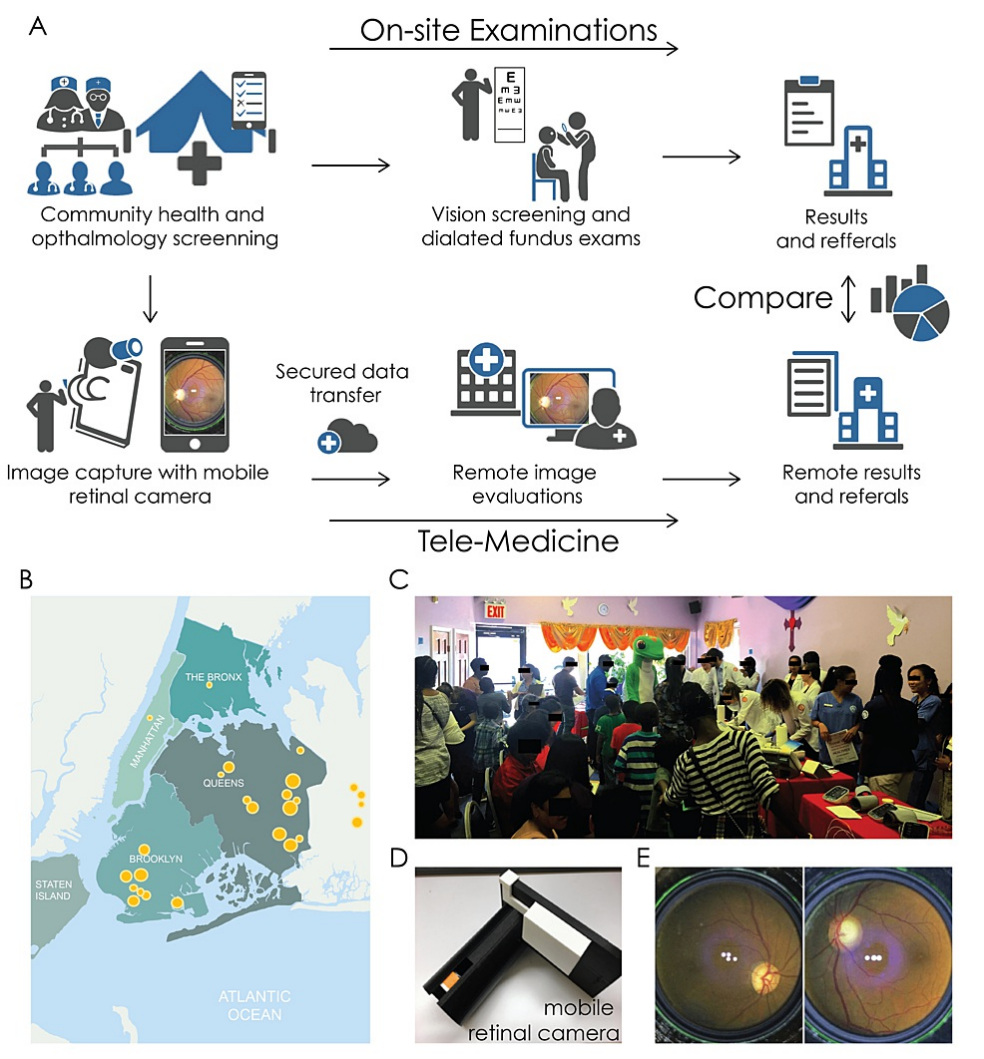
Screening events and data analyses method A) Diagram of the overall organization of this study. Participants provided health information and received a dilated exam; retinal images were obtained at the screening events and provided to remote ophthalmologists. The result from the dilatated exam by an attending physician was compared to the result from remote retinal image evaluation. B) Map of New York City indicating the locations of health screening sites. C) Example picture of a health screening event. This particular event was at a community church in Queens. D) The novel mobile retinal camera, designed for this study. E) Example images captured by the mobile retinal camera.

Community eye screening was conducted in partnership with an existing outreach program, Heart to Heart, hosted by the Weill Cornell Medicine Clinical & Translational Science Center (CTSC) (Figure 1). The study was approved by the Weill Cornell Medicine Institutional Review Board (IRB) with protocol number 1507016399. The Heart to Heart program screens members of the community for diabetes and heart disease at events at churches, community centers, and health fairs in low-income areas (Figures 1B, 1C). All participants undergoing cardiovascular screening through this group were offered the Eye to Eye screening, including dilated fundus exam and fundus photography. Medical student volunteers were recruited from all classes of medical students at Weill Cornell Medicine to perform ophthalmological screenings. Brief training was given to the students at the beginning of each event. An attending ophthalmologist or resident ophthalmologist was present at each screening event to oversee and conduct the on-site examination.

The screening included assessment of near and distance visual acuity, pupillary reflexes, extraocular movements, and intraocular pressure (measured with either iCare tonometer (iCare, Vantaa, Finland) or Tono-Pen tonometer (Reichert, Inc., Depew, NY), all performed by students under the supervision of an attending ophthalmology physician. All participants had retinal photographs taken with the mobile retinal camera (post-dilation) by trained students, followed by a dilated fundal exam with an indirect ophthalmoscope by an attending ophthalmologist. The fundal images were de-identified and were saved under a unique identification code for each participant. The on-site attending ophthalmologist additionally recorded an estimated cup to disc ratio for each eye and noted whether they recommended referral to an ophthalmologist. Patients with significant findings on the exam were referred to free and low-cost ophthalmological care resources provided by the Kress Vision Program.

We additionally had each participant fill out a survey on the intake that assessed baseline access to eye care in the form of previous interactions with eye professionals and patient-reported previous diagnoses. All findings, including demographics, social history, healthcare utilization, ocular history, family history, visual acuity, pupil exam, intraocular pressure, and significant exam findings, were recorded in a standardized form and collected through the REDCap secured database (Vanderbilt University, Nashville, TN). 

Novel retinal camera

We have developed a portable, low-cost system to capture images of the retina during a dilated exam. The mobile retinal camera was designed and produced by Du Cheng, one of the authors of this study. The principal components of the device include a 20D indirect ophthalmoscope (Volk, Mentor, OH), a smartphone (an iPhone 6S (Apple Inc., Cupertino, CA) in this case, an adjustable LED light source controlled by custom micro circuitry, and a custom device casing to assemble all the components in place. The digital file of the custom casing was generated and then produced by a MakerBot Method FDM 3D printer (MakerBot Industries, Brooklyn, NY) with Tough PLA materials (MakerBot Industries, Brooklyn, NY) with 200um layer resolution. The custom casing has a sliding slot on the side of the tube that holds the 20D indirect ophthalmoscope in place while allowing it to be adjustable for optimal focus. The custom circuitry includes a battery pack, switch, an adjustable resistor, and micro-SMD LEDs (Surface Mount Device Light Emitting Diode) (Evan’s Design, Collins, CO) that are embedded into the casing. The device is a class I device defined by the FDA 510(K) Exemption.

Evaluation of tele-health images

After each event, an independent resident ophthalmologist, blinded to the clinical information from the on-site examination, rated the de-identified smartphone-based and non-mydriatic fundal pictures based on the images alone. The images were first rated for quality, rated as ‘Good’, ‘Adequate’, or ‘Poor’. The images were then assessed for any significant clinical findings, including estimated cup-to-disc ratios. Recommendations for referral to further ophthalmology care were made based on the images as well. The on-site attending ophthalmologist’s evaluation was considered the “gold standard” for comparison to fundus photography of identification of optic disc cupping and the need for referral. Optic disk cupping was defined as a cup to disk (C:D) ratio greater than 0.5, and C:D ratio was reported for both observers. The overall workflow of the screening events and subsequent fundus image analysis is described in Figure 1A. 

Statistical analyses

Statistical analyses were performed using Microsoft Excel (Microsoft, Redmond, WA) and Prism 9 software (GraphPad, San Diego, CA), and graphs were generated in Microsoft Excel and Prism 9. For most analyses, summary statistics were utilized. These were computed in Microsoft Excel. Chi-Squared testing on overall vs study population demographics was performed in Prism 9. For the calculation of sensitivity and specificity of detecting optic disk cupping, we utilized the on-site ophthalmologist’s exam conclusion as the gold standard baseline. Sensitivity was calculated as: (#True Positives-onsite physician saw cupping and telemedicine physician saw cupping)/(#True Positives + #False Negatives-onsite physician saw cupping that was not observed by telemedicine physician), and specificity was calculated as (#True Negatives-onsite physician did not identify cupping and neither did the telemedicine physician)/(#True Negatives + #False Positives-telemedicine physician saw cupping that was not observed by an onsite physician). Sensitivity and specificity were calculated similarly for referral recommendation, with the onsite ophthalmologist’s recommendation considered to be the gold standard. We further characterized agreement between on-site and remote ophthalmologists using the weighted kappa statistic (kw), as previously defined [11,12]. Briefly, kw measures the chance of corrected agreement on a scale of -1.0 to +1.0, with -1.0 indicating perfect disagreement and +1.0 indicating perfect agreement, and 0.0 indicating no more agreement than would be expected by chance. For the C:D ratio, weights were applied as follows: 1.0 for a 0.0 difference, 0.95 for 0.15 difference, 0.9 for a 0.1 difference, 0.7 for a 0.15 difference, 0.5 for a 0.2 difference, and 0.2 for a 0.3 difference. A kw of 0.00 or less is poor, >0.00-0.20 is slight, 0.21-0.40 is fair, 0.41-0.60 is moderate, 0.61-0.80 is substantial, and 0.81-1.00 is almost perfect. 

## Results

Participant demographic information

**Figure 2:**
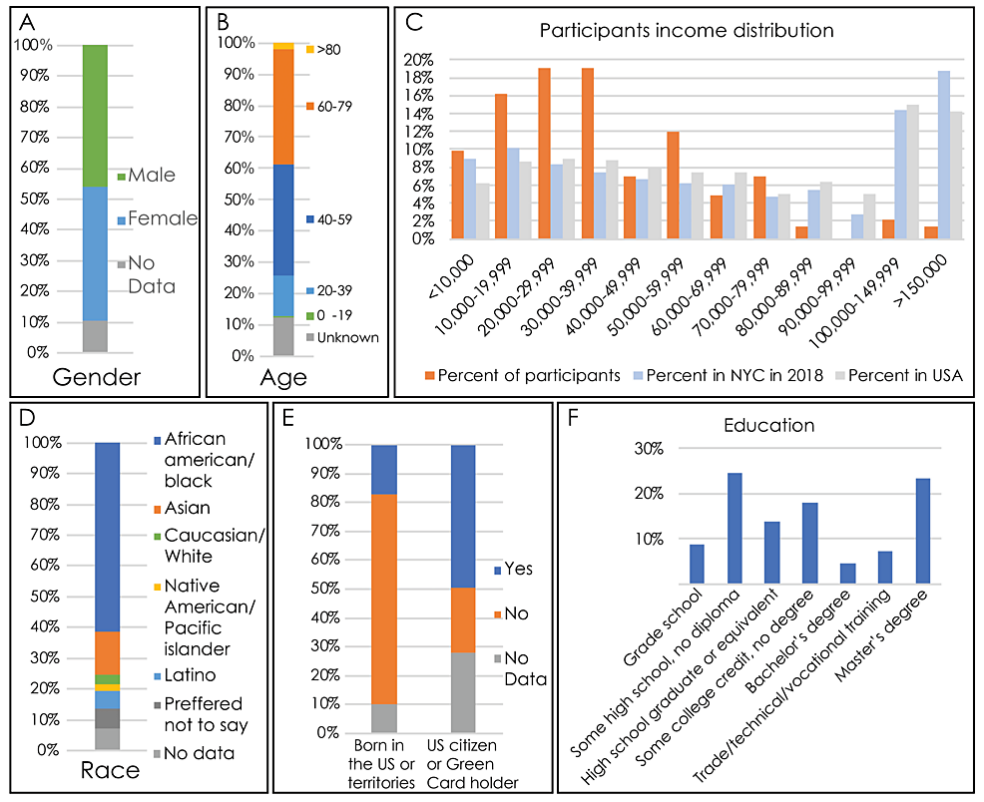
Demographic information of participants in the ophthalmology screening program A) Gender of the participants. B) Age of the participants. C) The income distribution of the participants compared to the income distribution of New York City and the United States. D) Race of the participants. E) Birthplace and citizenship status of the participants. F) Education status of the participants.

Participant demographics are shown in Table 1 (see appendix). A total of 112 community members underwent ophthalmological screening. Of the 96 participants who reported their sex, 44 were male and 52 were female (Figure 2A) ranging from 18-90 years old (Figure 2B). A total of 90 participants reported the zip code of their home address as well. A total of 34 participants were from Brooklyn, 48 from Queens, 6 from Long Island, 1 from Harlem, and 1 from the Bronx. The participants had a significantly different income distribution from both the New York City and the United States population as a whole (Chi-Squared test, p=0.0002). In the study population, the highest frequency of participants (25% of reported) had an annual household income between $30,000 and $39,999, followed by 18% in the $10,000-$19,999 bracket and 15% in the $50,000-$59,999 bracket. By contrast, the highest frequency income bin in New York City is >$150,000 (18.8%) and in the United States is $100,000 (15.00), respectively. One hundred of 112 participants reported their race, of which 60 were African American, 24 Asian, four Native American/Pacific Islander, three Caucasian, and two Latino (Figure 2D). Fifty-one participants reported being US citizens or green card holders. 82 reported that they were not born in the US or US territories, while 14 reported being born in the US or US territories (Figure 2E). In terms of participants' highest level of education attained, 15% of participants had a grade school education, 37% had education through high school, 16% held a bachelor’s degree, 4% had technical/vocational training, and 1% had master’s degree (Figure 2F). 

Participant ophthalmic characteristics

**Figure 3:**
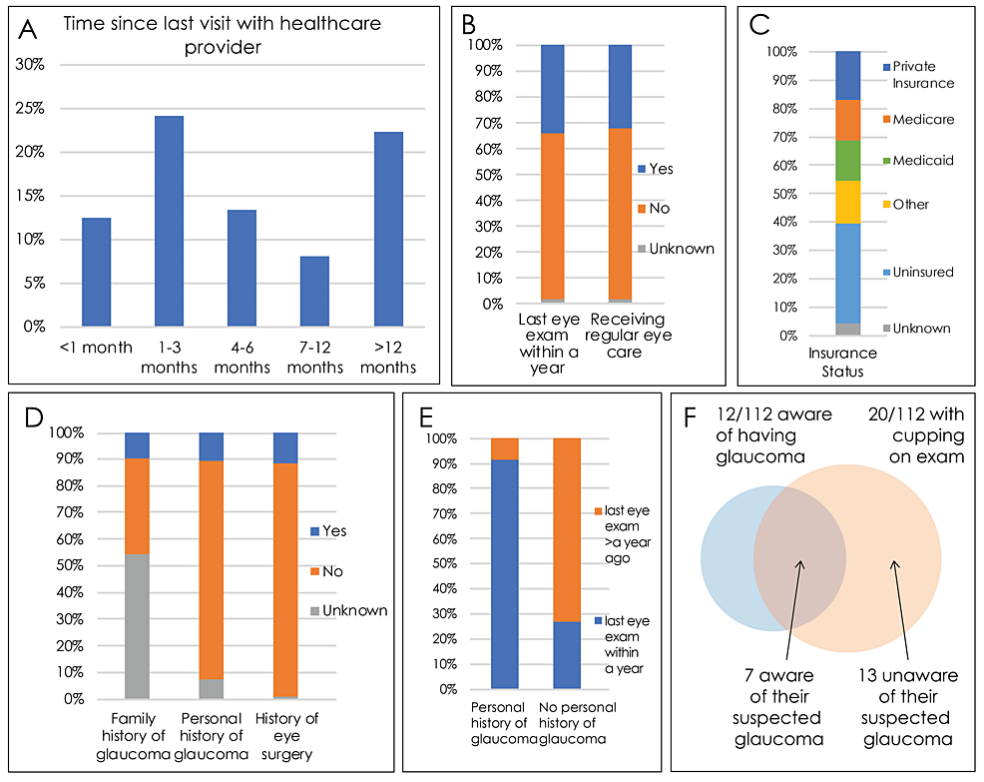
Overview of the health information of participants in the ophthalmology screening program A) Time since the participant last visited with a healthcare provider. B) Last time when the participant received an eye exam. C) Insurance status of the participant. D) Family history and surgical history of eye diseases of the participants. E) Time since the participant last received an eye exam, grouped by participants' medical history of diagnosed glaucoma. F) Venn Diagram illustrating the subset of the participants who were aware of their own disease status, of those found to have cupping on the exam. 13 out of 20 patients with optic disc cupping on exam were never aware of their possible glaucoma until the ophthalmology screening event.

Figure 3 gives an overview of the health information of participants in the ophthalmology screening program. Of the screening participants, 25% had not had a visit with a healthcare provider in >12 months (Figure 3A). 64% of the participants had not had an eye exam within a year and 66% were not receiving regular eye care (Figure 3B). A total of 35% of participants were uninsured, and the rest received insurance from Medicare (14%), Medicaid (14%), private (17%), and other/unknown sources (Figure 3C). 

Participants' ophthalmic exam results can be found in Table 2 (see in appendix). Visual acuity, measured in 224 eyes, was 20/20-20/25 in 123 eyes (55%), 20/30-20/50 in 84 (38%), 20/70-20/100 in 9 (4%), 20/150-20/400 in 3 (1%), and worse than 20/400 (counting fingers or light perception/hand motion only) in 5 (2%). Average intraocular pressure was 17 mmHg (range 10-28) in the right eye and 16.6 mmHg (range 8-30) in the left eye. Anterior segment findings in 214 eyes were normal in 140 (65%) and revealed cataracts in 36 (17%) and pterygium in 10 (5%). Eleven participants (10%) reported a family history of glaucoma, while 12 (11%) reported a personal history of glaucoma. Thirteen (12%) reported a history of eye surgery (Figure 3D). Of the 12 participants who had a history of glaucoma, 11 (92%) had an eye exam within the last year, while one had not. In 92 participants with no personal history of glaucoma, 25 (27%) had had an eye exam within the past year, while 53 had not and four did not know. Of eight participants with an unknown history of glaucoma, two had had an eye exam within the past year, while six had not (Figure 3E). Twenty patients had cupping on examination. Of these, seven were aware of their suspected glaucoma, and 13 were unaware. Five patients with a personal history of glaucoma or suspected glaucoma did not have cupping on examination (Figure 3F). 

Mobile retinal camera image quality

**Figure 4:**
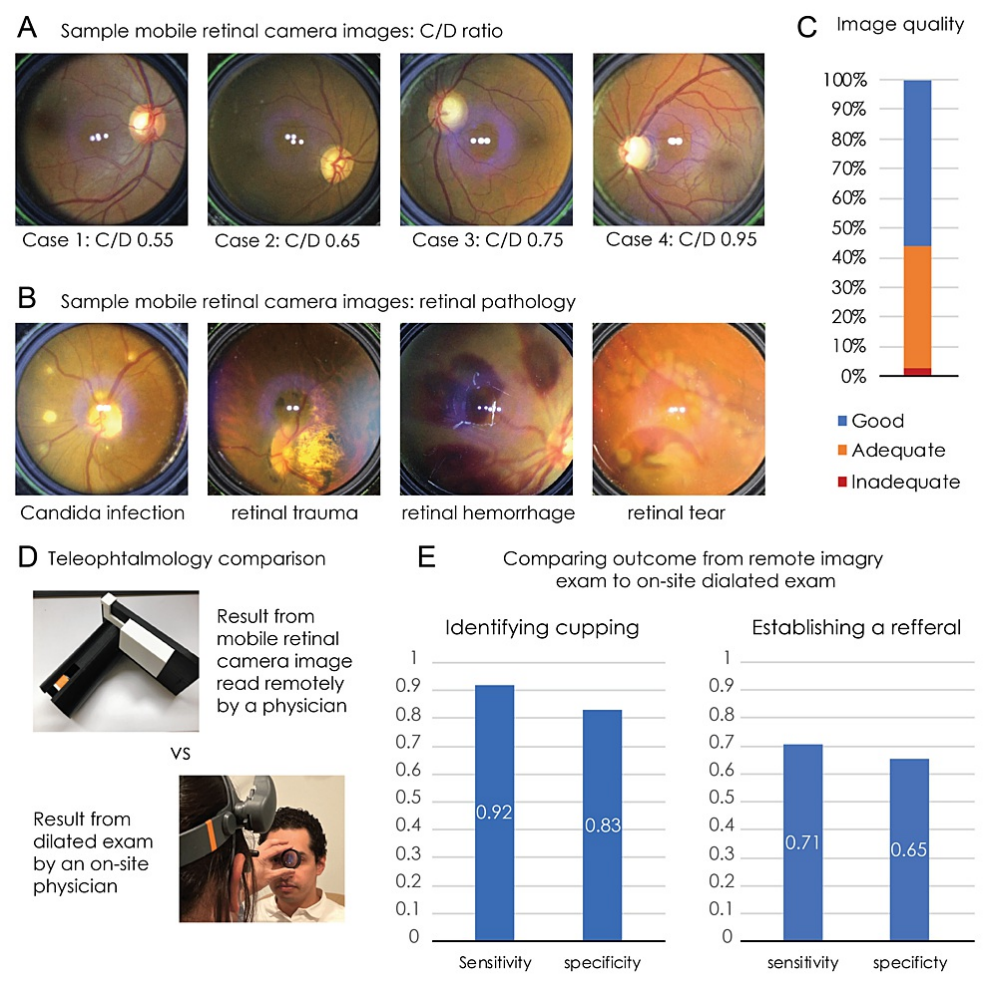
Efficacy of the novel retinal imaging device A) Pictures from example participant cases with varying C:D ratios from the mobile retinal camera B) Pictures from example participant cases showing varying retinal pathology from the mobile retinal camera. C) Image quality of the mobile retinal camera, rated by a clinician as either ‘good’, ‘adequate’, or ‘inadequate’. D) Image showing the set-up of a dilated eye exam comparing exam with an indirect ophthalmoscope to that with the mobile retinal camera. E) The sensitivity and specificity of identifying disc cupping (left) and the sensitivity and specificity of recommending a referral (right) by the remote evaluation of the mobile device image, compared to the dilated exam by an attending physician at the screening site as the “gold standard”.

Figure 4 presents the efficacy of the novel retinal imaging device. From the mobile retinal camera images, C:D ratios could be discerned (Figure 4A) and retinal pathology could be visualized (Figure 4B). 187 images were taken with the mobile retinal camera. Ophthalmologists graded 56% of the mobile retinal camera images as ‘Good’, 41% as ‘Adequate’, and 3% ‘Inadequate’ (Figure 4B). 

Efficacy of telehealth examination

The sensitivity of the remote images to detect cupping based on the reading of the in-person ophthalmologist was 92%, while the specificity for the agreement was 83% (Figure 4D). The on-site and remote ophthalmologists also evaluated participants regarding whether to refer for further care for 86 of the 112 patients. The on-site ophthalmologist recommended 34 participants of 86 total evaluated participants for further referral. The remote ophthalmologist recommended 42 of 86 total evaluated participants for further referral. The sensitivity of the remote images for generating referral recommendations was 71%, while the specificity was 65% (Figure 4D). 

## Discussion

Telehealth services have been gaining importance in disease monitoring, timely referral, and improving compliance with medical care [13,14]. Given the visual component of diagnosis, teleophthalmology may be both beneficial and feasible. Often ophthalmologic care is lacking in rural, remote, or underserved areas. The cost of physician-level care is often one of the largest components of medical care cost, another limiting factor [15]. Additionally, there is often a shortage of physicians with proper ophthalmologic training. For example, many countries have no neuro-ophthalmologists due to a lack of access to training [16]. Because ophthalmology is a visually intensive specialty, it is possible to have physicians assess pathology remotely [17]. Therefore, the ability to have students or other community centers perform screening and imaging reduces the need for physicians and has been used successfully in other locations [18,19].

Conventional retinal cameras are considerably larger and are generally not portable [10]. We have developed a novel ophthalmic telemedicine model using a mobile retinal camera that is portable and more affordable than existing devices. The mobile retinal camera is also user-friendly without formal ophthalmology training and equivalent or superior in performance to previously used imaging techniques at outreach events. The main limitation of the mobile retinal camera is the requirement for mydriasis for imaging. Our sensitivity and specificity of the remote physician’s findings in comparison to that of the attending at the screening site, as well as inter-ophthalmologist agreement on the C:D ratio indicate that the mobile retinal camera has potential for use in future teleophthalmology screenings. 

Our study is not the first to utilize remote image analysis. A pilot study in northern Manhattan, New York demonstrated a high prevalence of undiagnosed ocular pathology; however, our study differs in that screening images were collected by medical students, and we incorporated an on-site ophthalmologist to validate the accuracy of the telehealth recommendations [20]. The Philadelphia Telemedicine Glaucoma Detection and Follow-Up Study utilized a primary care telehealth ophthalmologic screening, followed by eye examination for those who needed it [21]. Interestingly, 17.1% (n=155/906) of images were rated as “unreadable” by the telehealth ophthalmologist, necessitating follow-up ophthalmologic examination to rule out ocular pathology [22]. Whereas in our study, only 3% of images were evaluated as “inadequate” by retina specialists. In a study done in South Africa, fundus images were transferred from an ophthalmoscope to a digital camera to a mobile phone to be sent via text message, and these remotely-acquired images were rated as acceptable for clinical use by ophthalmologists who received the images [8]. In China, ophthalmologic telehealth screening was provided by photos taken by a primary care provider and evaluated remotely by an ophthalmologist. Screening results were similar to those obtained by traditional methods [23]. However, these two international teleophthalmology studies of retinal disease screening did not demonstrate the efficacy of remote image-based screening through a direct comparison of the results to the on-site dilated exam. In this study, we demonstrated the efficacy of this device by high rates of sensitivity and specificity for identification of cupping and for generation of referral. When considering the onsite ophthalmologist’s exam and recommendations as the gold standard, the sensitivity and specificity for identification of cupping were 0.92 and 0.83, and the sensitivity and specificity for the follow-up recommendations were 0.71 and 0.65. These values are comparable with the sensitivity and specificity observed in other teleophthalmology programs [24]. Together, these data indicate that the performance of the mobile retinal camera is on par with existing technology in use in terms of sensitivity and specificity [25], as well as in terms of inter-rater reliability.

There is a need for affordable, easy-to-use methods of ophthalmologic screening in remote areas, given that current technology is both costly and bulky [26,27]. The mobile retinal camera costs less than $1000, compared with the conventional retinal cameras costing over $10,000. This mobile retinal camera is therefore low-cost compared to other currently used portable fundal imaging devices. The mobile retinal camera was also easy to use in the community setting for students with minimal training, as all screeners at community sites were medical students in various stages of training. The one drawback of the mobile retinal camera was that it required dilation. Nonetheless, while direct fundoscopic examination does not require dilation, dilation is commonly performed in most clinical settings before fundus and retinal examination, so the need for dilation prior to the use of the mobile retinal camera may not be a limitation to the utility of this device in most clinical settings. 

Importantly, our screening events reached a diverse population of New Yorkers, traditionally underserved by the medical community. Many were uninsured, disconnected from regular health care, and unaware of their ophthalmological risk factors or early signs of ophthalmological disease. That this study describes a screening that was provided in part by students using an affordable ocular imaging device adds to its benefit and applicability in additional clinical settings. This device and our findings may improve eye care in a population that is notoriously underserved by increasing diagnosis and recognition of eye diseases earlier in the disease course. 

The affordability and ease of use of this mobile retinal camera open doors for its use outside of ophthalmology settings, including use as an educational tool, a device for non-ophthalmologist physicians, and a source of images for patient education. At many institutions, medical students have little exposure to ophthalmology [28]. The availability of an affordable and easy-to-use device may increase the accessibility of ophthalmology for medical students who are earlier in their training. Furthermore, ophthalmoscopy is an important but often difficult skill for non-ophthalmology specialties, such as primary care providers [29]. As patients are often asymptomatic with respect to visual symptoms and may only be following up with a primary care provider, identifying all patients at risk for preventable causes of blindness requires fundoscopic examination outside of ophthalmologic settings. This user-friendly system may aid in the ease of other providers’ ophthalmologic exams. Finally, a previous study found that a lack of education about glaucoma in the community was more associated with poor follow-up than lack of access to care [30]. Of note, it has been demonstrated that showing patients their own retinal images can aid in their understanding of their diseases and possibly with treatment compliance [7]. Image capture by the mobile retinal camera during screening would provide an option for this method of patient education. 

## Conclusions

In conclusion, remote evaluation of images captured with an affordable, portable mobile retinal camera by medical trainees in community settings was effective in evaluating optic disc cupping compared to an attending physician's dilated fundus exam.

## Appendices

**Table 1:** Patient demographics and general health characteristics

Parameters	Number (%)
Total participants screened	112
Gender [n=96]	
Male	44 (46%)
Female	52 (54%)
Race [n=100]	
African American / Black	60 (60%)
Asian	24 (24%)
Caucasian / White	3 (3%)
Native American or Pacific Islander	4 (4%)
Latino	2 (2%)
Prefer not to state	7 (7%)
Born in the United States or US territories [n=96]	
Yes	14 (15%)
No	82 (85%)
US citizen or green card holder [n=80]	
Yes	51 (64%)
No	29 (36%)
Education [n=82]	
Grade school	11 (13%)
Some high school, no diploma	0 (0%)
High school graduate or equivalent	27 (33%)
Some college credit, no degree	12 (15%)
Bachelor’s degree	19 (23%)
Trade/technical/vocational training	3 (4%)
Master’s degree	10 (12%)
Income (dollars) [n=86]	
10,000-19,999	9 (10%)
20,000-29,999	6 (7%)
30,000-39,999	13 (15%)
40,000-49,999	4 (5%)
50,000-59,999	8 (9%)
60,000-69,999	2 (2%)
70,000-79,999	5 (6%)
80,000-89,999	1 (1%)
90,000-99,999	0 (0%)
100,000-149,999	2 (2%)
150,000 and greater	2 (2%)
Prefer not to report	34 (39%)
Time since last visit with healthcare provider [n=107]	
<1 month	14 (13%)
1-3 months	27 (25%)
4-6 months	15 (14%)
7-12 months	9 (8%)
>12 months	25 (23%)
Did not report	18 (17%)

**Table 2:** Ocular screening questions and examination findings

Ocular screening parameters	Number (%)
Family history of glaucoma [n=112]	
Yes	11 (10%)
No	40 (35%)
Unknown	61 (54%)
Personal history of glaucoma [n=112]	
Yes	12 (11%)
No	92 (82%)
Unknown	8 (7%)
History of eye surgery [n=112]	
Yes	13 (12%)
No	98 (88%)
Unknown	1 (1%)
Time since last eye exam [n=112]	
Within 1 year	38 (34%)
>1 year	70 (63%)
Unknown	2 (2%)
Receiving regular eye care [n=112]	
Yes	36 (32%)
No	74 (66%)
Unknown	2 (2%)
Visual acuity [n=224 eyes]	
20/20 to 20/25	123 (55%)
20/30 to 20/50	84 (38%)
20/60 to 20/100	9 (4%)
20/200 to 20/400	3 (1%)
worse than 20/400	5 (2%)
Tonometry	
Right eye	17.0 (range 10-28)
Left eye	16.6 (range 8-30)
Anterior segment (n=214 eyes)	
Normal	140 (65%)
Cataract	36 (17%)
Pterygium	10 (5%)
Not evaluated	28 (13%)
